# Recognizing the situation awareness of forklift operators based on EEG techniques in a field experiment

**DOI:** 10.3389/fnins.2024.1323190

**Published:** 2024-02-20

**Authors:** Xin Li, Yutao Kang, Weijiong Chen, Feng Liu, Yu Jiao, Yabin Luo

**Affiliations:** ^1^College of Ocean Science and Engineering, Shanghai Maritime University, Shanghai, China; ^2^COSCO SHIPPING Heavy Industry Co., Ltd., Shanghai, China; ^3^Merchant Marine College, Shanghai Maritime University, Shanghai, China

**Keywords:** situation awareness, EEG, correlation, recognition, forklift operators

## Abstract

Lack of situation awareness (SA) is the primary cause of human errors when operating forklifts, so determining the SA level of the forklift operator is crucial to the safety of forklift operations. An EEG recognition approach of forklift operator SA in actual settings was presented in order to address the issues with invasiveness, subjectivity, and intermittency of existing measuring methods. In this paper, we conducted a field experiment that mimicked a typical forklift operation scenario to verify the differences in EEG states of forklift operators with different SA levels and investigate the correlation of multi-band combination features of each brain region of forklift operators with SA. Based on the sensitive EEG combination indexes, Support Vector Mechanism was used to construct a forklift operator SA recognition model. The results revealed that there were differences between forklift operators with high and low SA in the *θ*, *α*, and *β* frequency bands in zones F, C, P, and O; combined EEG indicators *θ*/*β*, (*α* + *θ*)/(*α* + *β*), and *θ*/(*α* + *β*) in zones F, P, and C were significantly correlated with SA; the recognition accuracy of the model reached 88.64% in the case of combined EEG indicators of zones C & F & P as input. It could provide a reference for SA measurement, contributing to the improvement of SA.

## 1 Introduction

Forklifts are crucial engineering vehicles for industrial production. However due to the complicated environment of forklift operations and the numerous potential risks, the accident rate is very high globally (Marsh and Fosbroke, 2015). According to the U.S. Occupational Safety and Health Administration (OSHA), forklift accidents currently cause at least 85 fatalities, 34,900 serious injuries, and 61,800 minor injuries annually (Occupational Safety and Health Administration, 2021). According to Occupational Safety and Health Administration (2002–2021), 2,375 cases of forklift accidents were found, of which 816 were fatal, representing 34.36% of all forklift accidents. These statistic highlights the seriousness of forklift accidents. In many cases, forklift accidents can be attributed to human error on the part of the operator, such as inattention, misunderstanding or poor judgment (Sarupuri et al., 2016). In other words, forklift accidents are mainly caused by the forklift operator’s reduced situation awareness (SA) (Choi et al., 2020). Therefore, the level of SA possessed by forklift operators is a key factor in the safety of forklift operations, and accurate measurement of SA is essential for improving SA levels in forklift operation tasks.

Ensley (1995) first proposed the concept of SA, i.e., “the perception of elements in the environment within a volume of time and space, the comprehension of their meaning, and the projection of their status in the near future.” Most of the measurement methods of SA are based on Endsley’s three- level model and can be divided into Freeze probe techniques, Real-time probe techniques, Self-rating techniques, Observer rating techniques, and other types (Salmon et al., 2009).

Freeze probe techniques administer SA-related scales online during the “freeze” period of a task simulation and are used to “directly” assess participant’s SA. One of the most popular freeze probe techniques is the Situation Awareness Global Assessment Technique (SAGAT) (Endsley, 1995), which was developed to assess operator SA based on the Endsley three-level model. The main advantage of the freeze probe techniques is its direct and objective nature, which effectively solves some of the problems associated with post-test SA data collection (e.g., SA-performance correlation, poor recall, etc.).

Real-time probe techniques administer SA-related online scales without freezing the task. The Situation Present Assessment Method (SPAM) (Durso et al., 1998) is a typical real-time probe technique that was initially used to assess SA of air traffic controllers. Because no task freezing is required, the real-time probe technique is less intrusive compared to the freeze probe method.

Self-rating techniques usually involve subjective evaluation of the participant’s SA via a rating scale after the trial. The Situational Awareness Rating Technique (SART) (Taylor, 2017) is the most popular of these methods and measures the operator’s SA through 10 dimensions. The main advantages of self-rating techniques are their practicality and non-invasive nature.

The observer rating techniques involve domain experts observing participants during task performance and then assessing or rating each participant’s SA. The Situational Awareness Behavioral Rating Scale (SABARS) (Matthews and Beal, 2002) is a commonly used observer rating technique that has been used to assess the SA of infantrymen in field training. The observer rating technique has little to no effect on the task being performed and thus can be applied in realistic scenarios.

Nevertheless, the majority of SA measuring techniques now in use are intrusive, irrational, and intermittent, and they are unable to provide non-invasive and objective measures in non-interruptible situations, which compromises their ability to accurately estimate SA. Many studies in the field have shown that a variety of SA-related factors can be inferred in real time and indirectly utilizing physiological sensing techniques, i.e., employing physiological indicators like Electroencephalography (EEG) and eye movements to determine the operator’s SA level (Zhang et al., 2020).

EEG is the spontaneous rhythmic electrical activity generated by brain neurons recorded on the scalp (Proekt, 2018). According to the frequency of EEG in different human states, the frequency of EEG activity is usually divided into five types, i.e., *γ* wave, *β* wave, *α* wave, *θ* wave, and *δ* wave, which represent different human states (Teplan, 2002).

*γ* waves are high-frequency components of brain waves, most commonly found in somatosensory centers, and play an important role in cognitive activity of the human brain and in high-level activities such as information transfer, integrated processing and feedback in the brain. They generally occur when the brain performs cross-modal sensory processing tasks (e.g., synthesis of sound and light stimuli) or attempts to recall an object, and are sensitive to complex thinking operations (Wang et al., 2017).

*β* waves, with an amplitude of 5–20 *μ*V, are the faster frequency of brain waves and are more pronounced in the frontal and central regions. *β* waves represent the arousal of the brain, so it is generally believed that beta waves are the main electrical activity of the cerebral cortex in an excited state (Schubring and Schupp, 2019).

*α* wave amplitude is 20 ~ 100 μV, which is the faster frequency of brain waves. α wave can be detected in any part of human head, but it is more obvious in occipital and parietal regions, and its shape is similar to a sine wave. *α* wave is the basic brain wave in normal human, and its frequency is very constant when there is no external stimulation. It is generally believed that alpha waves are the main electrical activity of the cerebral cortex in the awake, quiet state (Pfurtscheller et al., 1996; Lim et al., 2014).

*θ* waves have an amplitude of 20–150 *μ*V and are the slower of the brain waves, generally more pronounced in the parietal and temporal regions. Typically, *θ* waves are not recorded in the waking state of healthy adults, but can be recorded only in the sleep state, especially when frustrated and depressed (Harmony et al., 1996; Sammer et al., 2007).

*δ* waves are generally of relatively large amplitude, about 20–200 *μ*V, and are the slowest of the brain waves, occurring primarily in the frontal and occipital lobes. Typically, *δ* waves are not recorded in the waking state in healthy adults, and they appear only in the presence of deep anesthesia, deep sleep, hypoxia, extreme fatigue, or organic brain lesions. Therefore, it is generally believed that *δ* waves are the main electrical activity of the cerebral cortex in the inhibited state (Münch et al., 2004).

These EEG indicators correlate with a person’s mental state and are commonly used to assess mental workload, fatigue and drowsiness (Borghini et al., 2014). Moreover, studies have been conducted to reveal the relationship between EEG signals in different frequency bands and cognitive workload (Puma et al., 2018; Iqbal et al., 2020; Jao et al., 2020), and to classify and assess cognitive workload on this basis (Mahmoud et al., 2017; Zhou et al., 2021). In addition, studies have also combined EEG spectral features according to different algorithms for assessing mental fatigue (Jap et al., 2009, 2011; Chen et al., 2013), where *α*, *β*, and *θ* waves are the frequency bands often used in combination.

Many studies have verified the correlation between EEG activity and SA in different domains. The use of EEG to measure SA has great potential (Lee et al., 2017). Catherwood et al. (2014) identified brain regions that showed strong activity during SA loss and measured EEG activity throughout the simulated task, but the study did not address the band characteristics of the subjects. Berka et al. (2006) revealed a correlation between certain brain frequencies and SA, however, the study did not find correlations between participants’ brain regions and SA. Fernandez Rojas et al. (2019) used EEG in a teleoperated system to predict different levels of SA. It was found that *θ*, *α*, and *β* bands present an increase during SA loss, and frontal and occipital regions are identified as reflecting changes in SA. Kaur et al. (2020) used the Stroop test to assess the effect of EEG pre-task rest information on SA and showed that Pre-task Resting Absolute Alpha (PRAA) and Pre-task Resting Alpha Frontal Asymmetry (PRAFA) were associated with performance on subsequent SA tasks when the task was subjected to the Stroop effect. Liu et al. (2023) found that SA level 1 and 3 were significantly correlated with some EEG signals, which were mainly *δ* and *θ* band power, but there was no correlation between SA and the α and β band power. And there was no significant correlation between SA level 2 and all EEG signals. Therefore, the identified electrode channels were mainly from the frontal and parietal regions, which are the core cognitive resources in the SA comprehension phase.

Few studies have further explored the identification of SA based on EEG spectral features. Yeo et al. (2017) applied machine learning methods to predict SA in air traffic controllers using EEG band metrics *α*, *θ*, *β*, entropy and band power ratio. It was found that support vector regression using EEG signals had the lowest prediction error of 1.5, outperforming the results of predicting SA using linear regression and extreme learning machine (ELM). Kästle et al. (2021) used a 32-channel dry EEG to acquire EEG data from 32 participants who completed the SA test in the Psychology Experiment Building Language (PEBL) task, determined the correlation between *β* and *γ* band signals in the left and right parietal and temporal regions of the brain and SA levels in the participants, and used Random Forest (RF) and Boosted Trees to identify SA in the participants with a maximum accuracy of 67%. Recently, Yiu et al. (2022) proposed a human-center approach to identify potentially hazardous weather conditions from EEG power spectral densities with Bayesian neural networks (BNN). The classification model has outperformed other baseline algorithms with an accuracy of 66.5%. Feng et al. (2022) used SA-sensitive EEG features fed into principal component analysis (PCA) and the Bayes method to discriminate different SA groups, and the accuracies were 83.3% for the original validation and 70.8% for the cross-validation. Jiang et al. (2023) developed an RF algorithm by PCA on EEG features with significant correlation with SA for further feature combination, which was then fed into CNN classification algorithm to obtain a classification recognition accuracy of 84.8%.

The aforementioned findings offer support for the application of EEG in cognitive assessment and offer a reference index for physiological EEG measurements in SA. However, there is no consensus on which EEG indicators differ significantly from SA in different research domains and which EEG indicators can be used to assess SA. And relatively few studies have further explored the detection of SA based on EEG spectral features. In addition, there is no precedent for investigating the relationship between SA and EEG feature metrics in forklift drivers. As an expansion and development of earlier studies, this study seeks to present a method for EEG identification of SA in forklift operators in real-world settings, providing an important reference for physiological measures of SA in forklift operation tasks.

In this study, we design and conduct a forklift operating field experiment, use Bitbrain semi-dry EEG to collect EEG data, and use the SAGAT method to assess the SA levels of the participants. Based on these experimental data, we first examine whether there are differences in the EEG spectral characteristics of forklift operators with different SA levels (RQ1). Subsequently, we aim to validate the relationship between SA scores and combined EEG indicators and to find out which indicators are significantly correlated with SA (RQ2). Finally, we construct a forklift driver SA recognition model based on these combined EEG indicators that are significantly correlated with SA (i.e., sensitive EEG indicators), and investigate the validity of SA recognition based on sensitive EEG indicators through model performance evaluation (RQ3).

RQ1. Are there differences in the EEG spectral characteristics of forklift operators with different SA levels?

RQ2. What are combined EEG indicators significantly correlated with SA scores?

RQ3. How can forklift operators’ SA be identified based on sensitive EEG indicators?

## 2 Materials and methods

### 2.1 Experimental design

#### 2.1.1 Participants

Fifteen forklift operators were recruited for this study. Since this experiment was conducted outdoors, it may be affected by the environment as well as the limitations of the equipment itself, resulting in missing data collection. Therefore, 11 participants with a data collection rate of more than 80% were used as participants for the study. These participants were males with normal vision who had between 2 and 27 years (*M* = 11.73, *SD* = 9.00) of practical experience operating forklifts between the ages of 30 and 53 (*M* = 49.75, *SD* = 2.22) and had no history of mental illness, brain disease, etc. Aside from having essentially the same driving experience on the experimental location and being capable of completing all driving tasks on their own, all participants had successfully completed field driving training prior to the experiment. Before the experiment, all participants provided written informed consent after being told of its details.

#### 2.1.2 Experimental equipment

**Figure 1:**
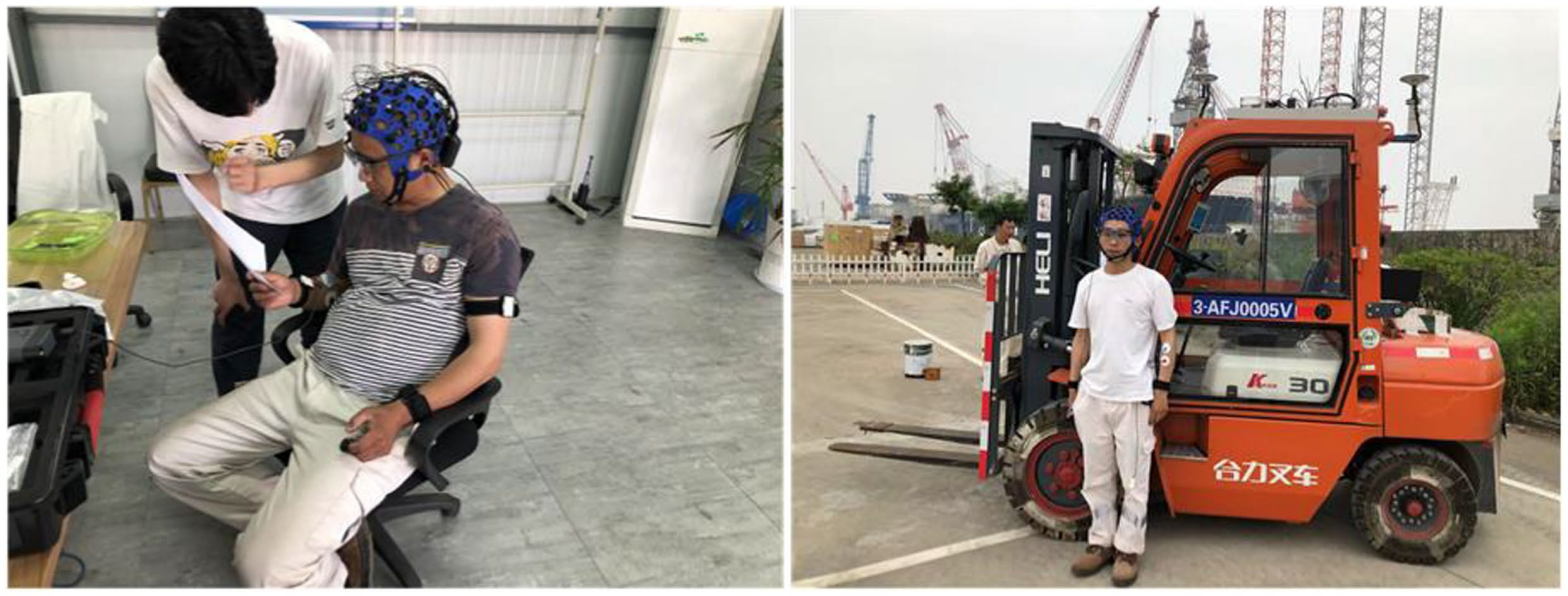
Experimental equipment.

As seen in Figure 1, the mechanical internal combustion counterbalanced forklift HELI CPC30 with a weight of 3 tons was employed in the experiment. Since it is closer to the actual production vehicle, this kind is the one chosen by the majority of national licensing agencies, training institutes, and competition organizations at all levels for training, testing, and competition.

Given the objectives and environmental conditions of the study, the semi-dry Bitbrain EEG based on water electrodes was chosen to obtain EEG signals from participants in real time during the experiment. The Bitbrain semi-dry EEG is a mobile EEG device that can dynamically record EEG activity in real time, mainly composed of 32-channel electrode caps, signal amplifiers and wires, and the sampling frequency was set at 256 Hz, as shown in Figure 1. Bitbrain is designed for multi-modal monitoring in a variety of simulated or real environments with maximum freedom of movement for the participant. EEG electrode caps comply fully with international standards for the potential distribution of common systems and are equipped with very stable contacts and active protection, allowing a relatively reliable and accurate monitoring in mobile conditions or in the presence of electromagnetic noise.

**Figure 2:**
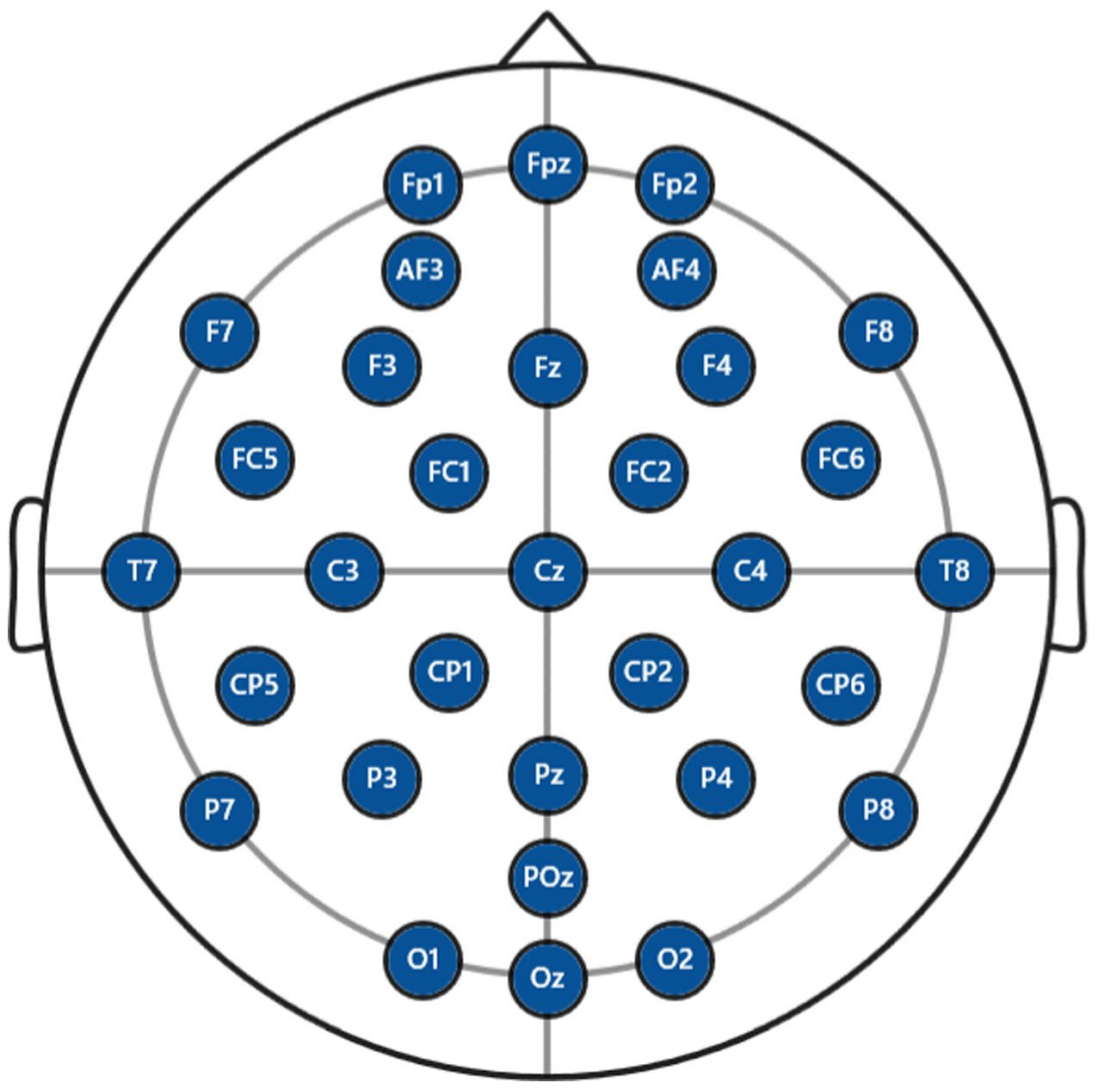
Standard position of EEG electrode. F, frontal lobe; C, central area; P, parietal lobe, T, temporal lobe; O, occipital lobe. The odd and even numbers correspond to the left and right sides of the brain area, respectively. The suffix p is used only for the prefrontal area, e.g., Fp1 indicates the left side of the prefrontal area and Fp2 indicates the right side of the prefrontal area.

In the actual recording of the EEG, it is necessary to determine the position of EEG electrode in order to reflect the corresponding neuronal activity in different regions of the brain. According to the international unified standard, the brain has five main regions: the frontal, central, parietal, temporal and occipital lobes, respectively, marked by the prefixes F (frontal lobe), C (central area), P (parietal lobe), T (temporal lobe), and O (occipital lobe), as shown in Figure 2. Among them, the odd and even numbers correspond to the left and right sides of the brain area, respectively, and the suffix p is used only for the prefrontal area, e.g., Fp1 indicates the left side of the prefrontal area.

#### 2.1.3 Experimental scenario

The forklift experiment is carried out at a typical forklift test site of a shipbuilding enterprise to make sure the experiment is acceptable and compliant and to better control variables. The test site is a typical location constructed in accordance with national special equipment operating license and forklift operation test standards that can offer all test elements and perform hands-on operation, simulation training, and training evaluation under the majority of forklift driving scenarios.

**Figure 3:**
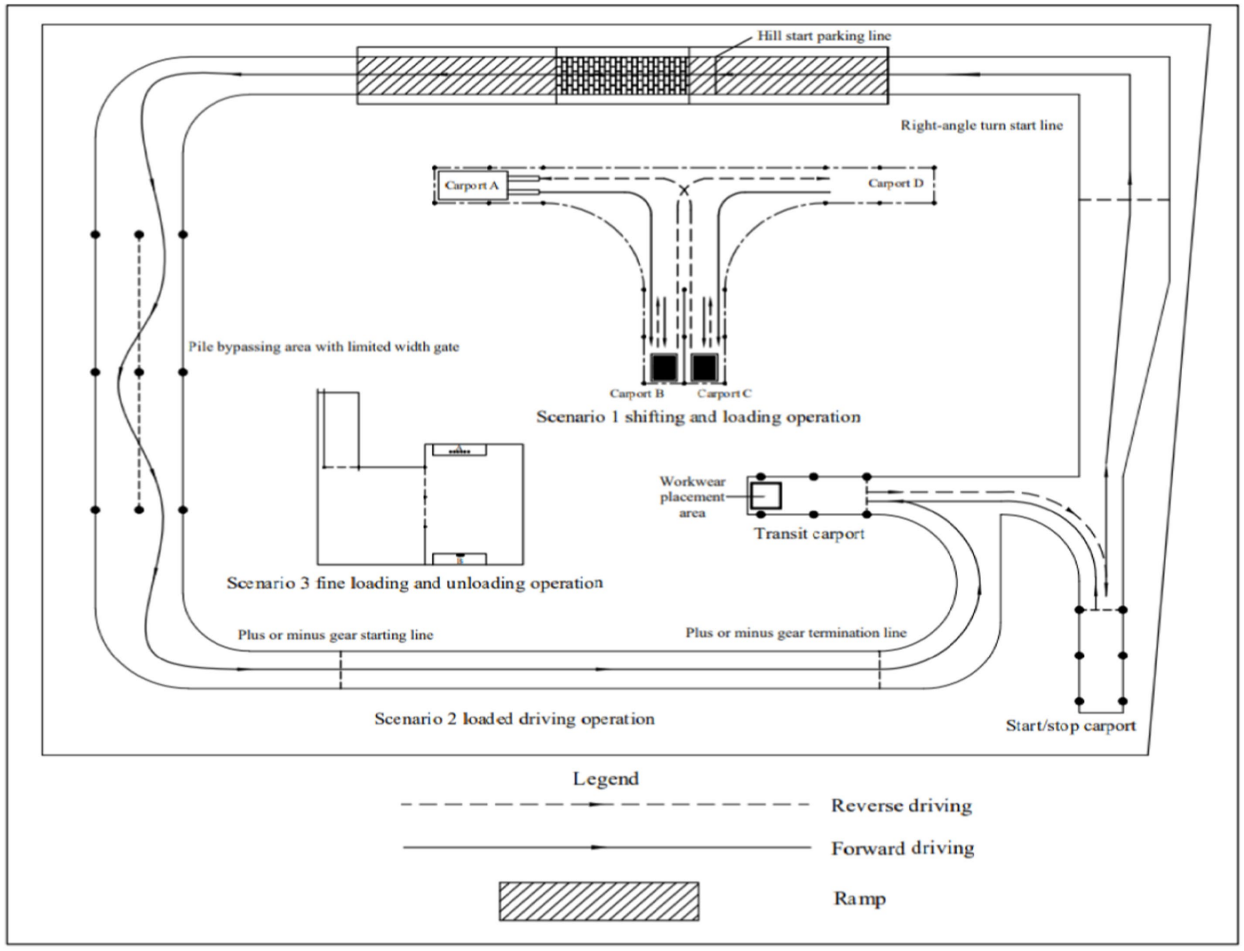
Experimental scenarios.

The experiment is based on the practical test of a national special equipment for the operator of forklift trucks, and considering that 80% of forklift truck accidents occur during the driving and operation, three typical scenarios related to the actual operation of forklift trucks are selected, including shifting and loading operation (Scenario 1), loaded driving operation (Scenario 2) and fine loading and unloading operation (Scenario 3). From scenario 1, the participants first completed the loading and unloading operation; then, in the special test area with curves, ramps, factory traffic signals and long distances (Scenario 2), completed the load driving operation of starting, moving, steering, ramp stopping and starting, turning, stopping, etc.; finally, completed the fine loading and unloading operation in scenario 3. All participants of the experiment had the same driving scenario and the same operating tasks in the same scenario. The experiment required the participants to perform the operational tasks of the three scenes continuously, and the driving route of the forklift is shown in Figure 3.

#### 2.1.4 Experimental protocol

Before the experiment, the participants were taught safety training for the driving of forklifts and informed of the detailed experimental procedures and operational tasks. The participants were also obliged to complete a basic statistical information questionnaire, including questions on age, occupation, years of license to drive a forklift, and real years of forklift driving. Participants wore an EEG device, an EEG signal acquisition device, with the help of the researcher. The ErgoLab software was then used for calibration and adjustment.

In the experiment, the participants performed three typical forklift operation scenarios according to the previously predefined driving route and completed the operation task for approximately 20 min. EEG data were recorded from 32 potentials in the F, C, P, T, and O brain regions during the experiment, and the EEG signals were synchronized and analyzed and processed through the ErgoLAB human-machine-environment synchronization cloud platform after the data acquisition was completed to obtain the frequency band index data.

**Figure 4:**
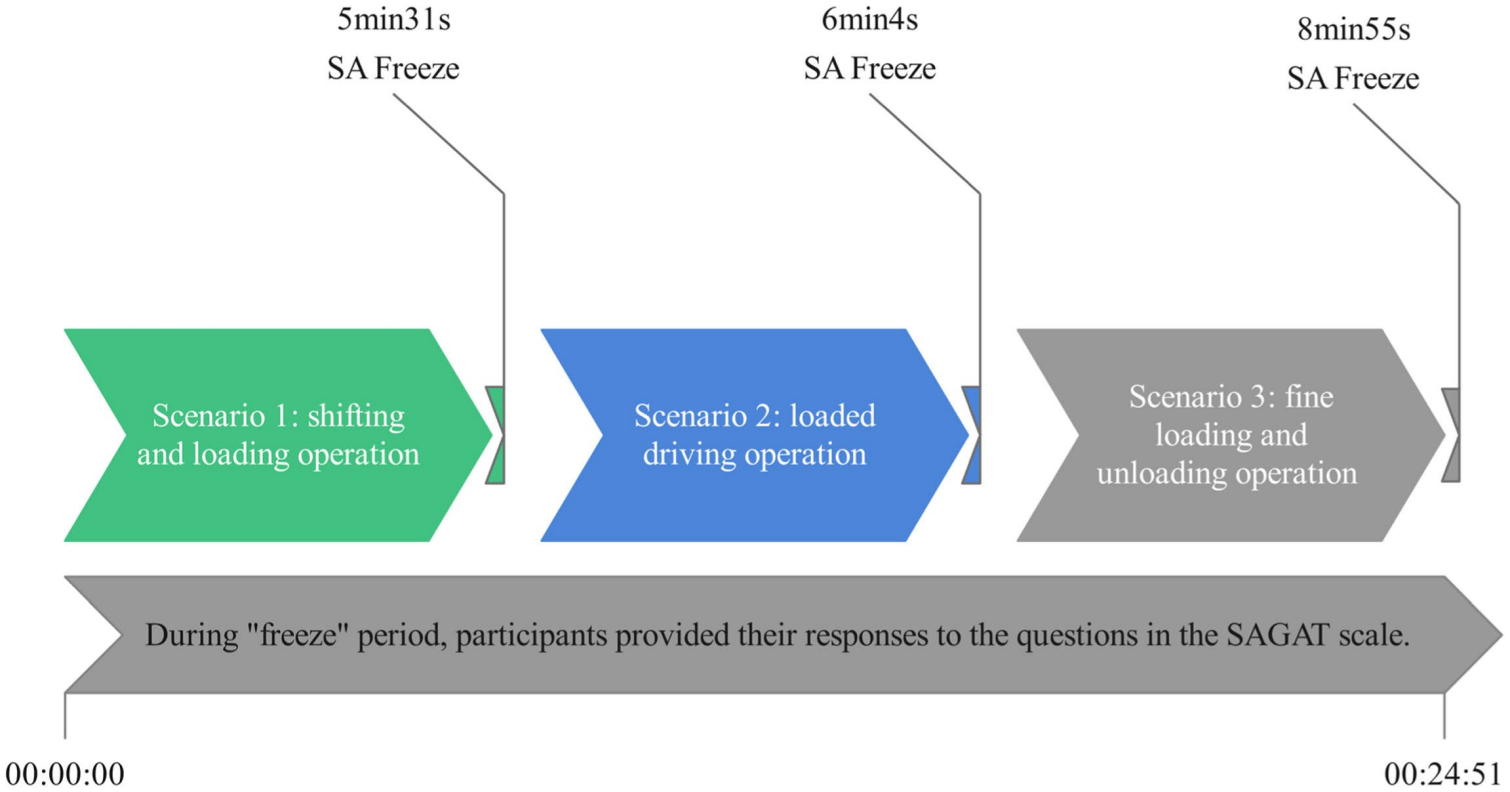
Procedures for measuring participants’ SA by the SAGAT method. There are a total of three “freezes,” one after each scenario task. SAGAT, Situation Awareness Global Assessment Technique; SA, Situation Awareness.

**Figure 5:**
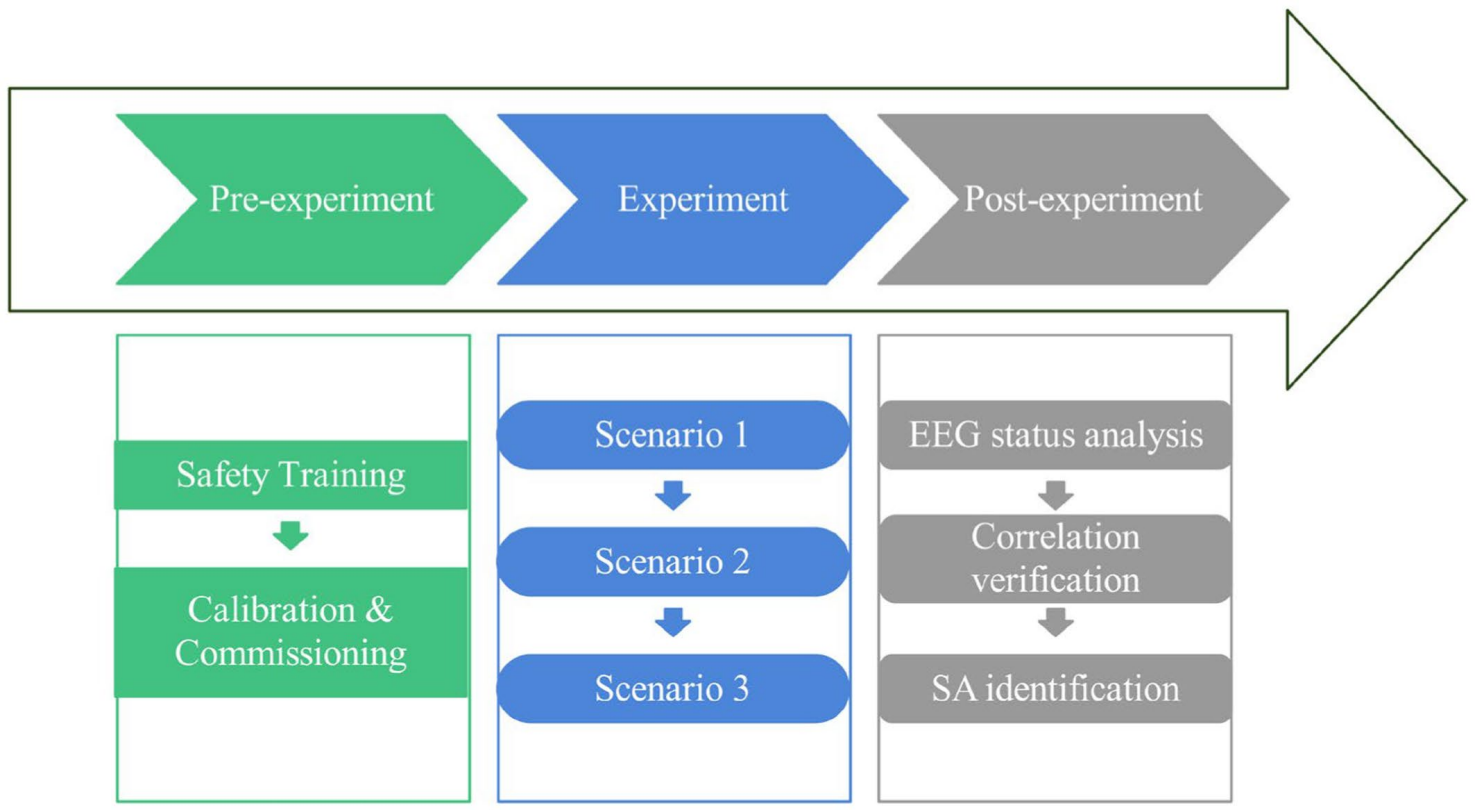
Experimental protocol.

After completing each scenario task, a questionnaire was administered with the SAGAT scale to assess the SA level of the participants in the forklift operation simulation experiment, as shown Figure 4. The SAGAT questionnaire was listed in Table 1. During “freezes” period, the participants’ SA levels were further confirmed by communicating briefly with them about their operating behaviors and mental activities during the forklift operation. The complete experimental protocol is shown in Figure 5.

**Table 1:** The SAGAT questionnaire.

SA levels	Related questions	Scoring criteria
Perception	The information I need to assess driving safety is readily available.	Extremely easy extremely difficult 7 score---------------------- 1 score
	I often forget about the safety information that keeps me safe while driving.	Frequent never 7 score---------------------- 1 score
	There are situations where control is lost due to receiving too many messages simultaneously.	Many minimal 7 score---------------------- 1 score
	I have encountered security messages that are often difficult to comprehend.	Many minimal 7 score---------------------- 1 score
Comprehension	I am aware of the dangers of driving.	Extremely understand extremely unclear 7 score----------------------- 1 score
	I am confident that I can handle any adverse event that may occur while driving.	Extremely confident extremely unconfident 7 score---------------------- 1 score
	I am aware of the practices that keep safe while driving.	Extremely understand extremely unclear 7 score----------------------- 1 score
	I possess a comprehensive understanding of the information that is and is not pertinent to safe driving.	Extremely understand extremely unclear 7 score----------------------- 1 score
	I am fully conscious of the safety implications of every action I take while driving.	Extremely understand extremely unclear 7 score----------------------- 1 score
Projection	I frequently notice potentially hazardous situations that may arise in the future.	Frequent never 7 score---------------------- 1 score
	I can anticipate negative events that may occur while driving.	Frequent never 7 score---------------------- 1 score
	I am aware of the emergencies that can occur when a dangerous situation arises while driving.	Extremely understand extremely unclear 7 score----------------------- 1 score
	I understand the consequences of hazardous driving situations.	Extremely understand extremely unclear 7 score----------------------- 1 score

SAGAT, Situation Awareness Global Assessment Technique; SA, Situation Awareness.

### 2.2 Data acquisition and preprocessing

Noise such as ECG, blinking, EMG, etc. can readily interfere with the acquisition of EEG data. The raw EEG data must therefore be cleaned of noise interference. High-pass, low-pass, and band-stop filters are frequently applied to the EEG signal. The EEG signal that was recorded for this experiment has a frequency range of 1–60 Hz. To eliminate noise interference from the raw EEG data, the filtering process is conducted using the ErgoLAB physiological analysis module in this study. The low-pass filtering stop frequency is established at 80 Hz to eliminate high-frequency signals, such as EMG artifacts. Similarly, the high-pass filtering stop frequency is set at 1 Hz to remove low-frequency signals, such as oculo-electrical artifacts. Additionally, the center frequency of the band-stop filtering is set at 50 Hz to eliminate utility interference.

### 2.3 EEG status analysis

The pre-processed EEG data were subjected to power spectral density (PSD), brain topography, and time-frequency analyses in order to dynamically reflect the EEG activity in each brain region frequency band. These analyses were combined with the SAGAT results to confirm the differences in the EEG signals of forklift drivers with various SA levels. This serves as the foundation for the subsequent correlation analysis and SA identification model construction.

#### 2.3.1 PSD analysis

A prominent method for examining EEG frequency domains is PSD analysis. It is intended to translate the EEG amplitude against time into an EEG power versus frequency spectrum so that the distribution and evolution of EEG rhythms can be seen graphically. The frequency in Hz is typically on the horizontal axis of the power spectrum density diagram, with the energy in dB on the vertical axis, allowing for visual examination of the distribution of energy values in various frequency bands and identification of the most active frequency band.

#### 2.3.2 Brain topography analysis

Based on the energy values of each electrode site at various frequency bands, brain topography is an energy spectrum. Based on the topography of the brain, it is feasible to visually examine which frequency band and which area of the brain are more active at a given time.

#### 2.3.3 Time-frequency analysis

To obtain information about EEG frequency fluctuation over time, time-frequency analysis applies the Fourier transform or wavelet transform to the raw EEG data. The results are displayed in a time-frequency diagram. Each time point on the time-frequency diagram corresponds to a power value, with time acting as the horizontal axis and frequency as the vertical axis. On the basis of the time-frequency diagram, it is feasible to visually assess how the EEG’s energy changes across various frequency bands as time passes.

### 2.4 Correlation test methods

Based on the results of the above EEG state analysis and combined with the findings of EEG-related literature, the commonly used EEG combination indicators affecting cognitive function, namely *α*/*β*, *θ*/*β*, (*α* + *θ*)/*β*, (*α* + *θ*)/(*α* + *β*), and *θ*/(*α* + *β*), were selected as candidate indicators. To investigate the correlative effect between EEG status and SA of forklift drivers, hypothesis testing was used to examine the correlation between combined EEG indicators and SA levels. Then, to further test the validity of the analysis results, principal component analysis combined with LASSO regression method, i.e., sparse principal component analysis, was used to calculate the characteristic index weights. Finally, the EEG combination indicators significantly correlated with SA were extracted by comparing the overlap of the correlated EEG indicators obtained by hypothesis testing with the indicators with higher weights obtained by PCA + LASSO calculation.

### 2.5 SA recognition model

Support vector machine (SVM) is one of the frequently employed machine learning techniques to handle binary classification problems, and the recognition of SA level for forklift drivers is essentially a binary classification problem. Therefore, in this paper, SVM is used to build the recognition model of SA level for forklift drivers, and the effective recognition of SA under forklift operation task is realized after training, testing, and performance evaluation of the model. This is based on the multi-band combination indices of each brain region significantly related to SA obtained in the previous chapter.

A more accurate classification is made possible by the toolbox version 3.23 created by Chang and Lin (2011), a researcher at NTU, which uses SVM as the core and introduces a third-party optimization parameter algorithm. This is because the basic SVM’s classification performance is easily constrained by the number of samples.

The main factors influencing the SVM model’s capacity for learning and generalization are its kernel functions and parameter selection. Kernel functions can be classified as one of four categories: linear, polynomial, gaussian (RBF), and sigmoid. Among these, the RBF kernel is a popular kernel function that has the advantages of large mapping dimensions, diversified decision limits, and consistent performance. It can map samples to a high-dimensional space and thus handle the nonlinear relationships between class labels and characteristics. Moreover, RBF has a low level of computational complexity. RBF was therefore selected for this paper’s SVM kernel function.

EEG feature indicator data training data set T = {(x1,y1), (x2,y2), …, (xN,yN)}, where $x_{i}=F\frac{\theta }{\beta },\;F\frac{\alpha +\theta }{\alpha +\beta },\;\cdots P\frac{\theta }{\alpha +\beta }\text{,}$ $x_{i}\in R^{n}$ is the i-th EEG feature indicator; $y_{1}\in \left\{0,1\right\}$ is the SA level classification label of the ith EEG feature indicator, where 0 indicates low SA level and 1 indicates high SA level; i=1,2,3⋯N , and N is the sample size.

First, by selecting an appropriate kernel function K(x,z) and penalty parameter C > 0, the convex quadratic programming problem is constructed and solved, as shown in Equations (1–2).


(1) $$\min_{\alpha }\frac{1}{2}\sum_{i=1}^{N}\sum_{j=1}^{N}\alpha_{i}\alpha_{j}y_{i}y_{j}K\left(x_{I},x_{j}\right)-\sum_{i=1}^{N}\alpha_{I}$$

(2) $$s.t.\sum_{i=1}^{N}\alpha_{i}y_{i}=0\text{,}\mathrm{where},0\leq \alpha_{i}\leq C,i=1,2\cdots N$$

The optimal solution $\alpha^{*}={\left(\alpha_{1}^{*},\alpha_{2}^{*},\cdots \alpha_{N}^{*}\right)}^{T}$ is obtained.

Also, under the condition that the RBF kernel is used as the kernel function, the classification decision function is shown in Equation (3).


(3) $$F\left(x\right)=\mathrm{sign}\left\{\sum_{i=1}^{N}a_{1}^{*}y_{i}\exp\left(-\frac{\left\|x-z^{2}\right\|}{2\sigma^{2}}\right)+b^{*}\right\}$$

Then, two important parameters, penalty parameter C and kernel function parameter gamma, need to be optimized, which is an important step in training the SVM model. The penalty parameter C and the kernel function parameter gamma are the core parameters of the SVM model, which can improve the training efficiency of the SVM. These two parameters are generally determined empirically during the modeling process or by conducting experiments in a certain range.

Grid search is a simple and intuitive optimization algorithm that can identify the best combination within a given hyperparameter space. In this study, grid search was employed to explore the optimal penalty parameter C and kernel function parameter gamma, and cross-validation techniques were utilized to enhance the accuracy of the training set classification. Initially, grid search sets a candidate set of values for each hyperparameter, then generates the Cartesian product of these candidate values to form a grid of hyperparameter combinations. Subsequently, grid search trains and evaluates the model for each hyperparameter combination, thus identifying the best-performing hyperparameter combination.

## 3 Results

### 3.1 SA rating

**Figure 6:**
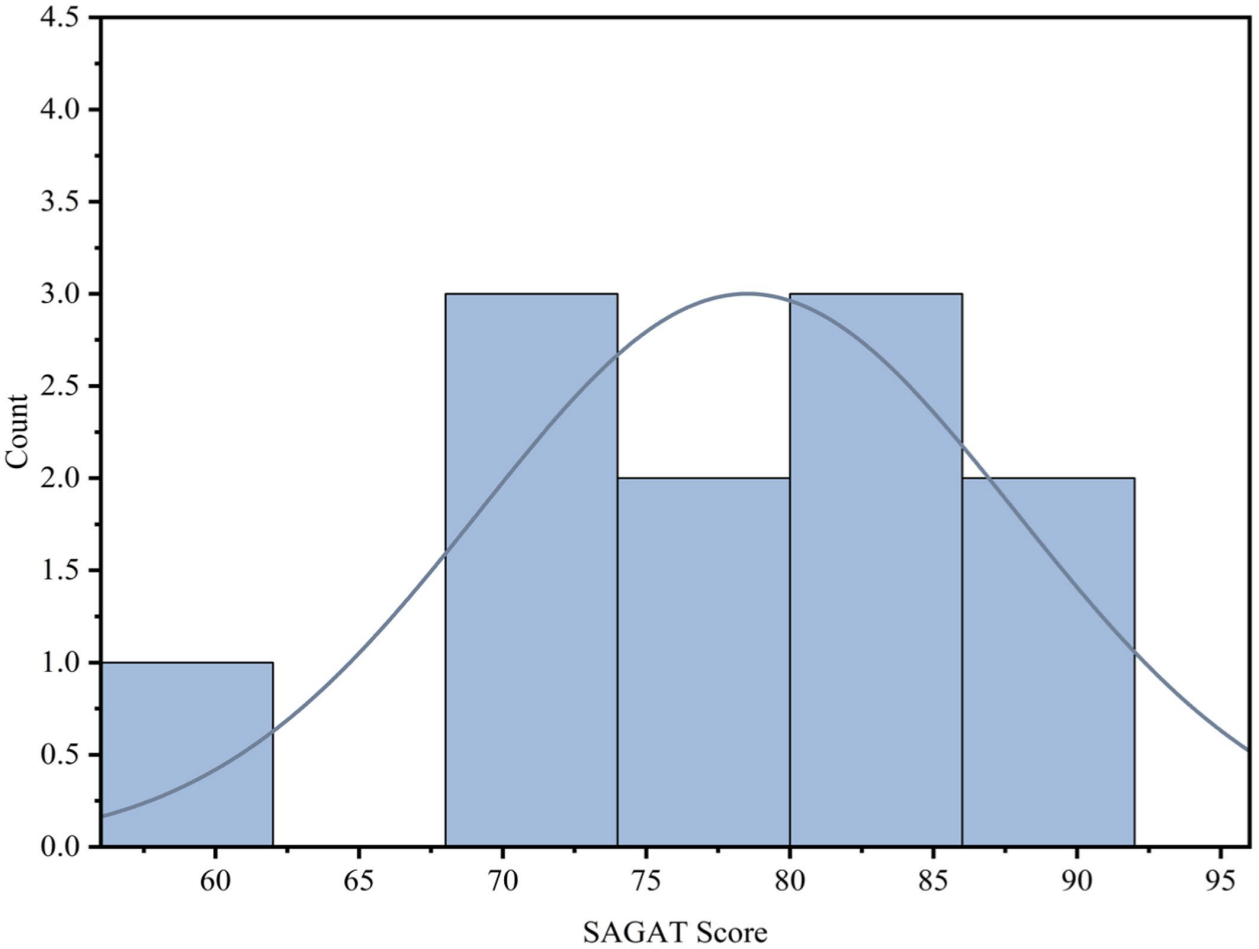
Distribution of the SAGAT scores for the participants. SAGAT, Situation Awareness Global Assessment Technique.

Based on the survey results from the SAGAT questionnaire, the SA score for each participant was calculated, and the distribution (Mean = 78.52, SD = 2.19) is shown in Figure 6. As the SA scores exhibit a normal distribution pattern, the mean SA score was used as the criterion to categorize participants into two groups: those with SA scores higher than the mean were classified as the high SA group, while those with scores lower than the mean were classified as the low SA group. The classification results indicated that participants 2 (72.33), 3 (69.33), 5 (73.33), 7 (60.67), 8 (76.14), and 9 (76.33) belonged to the group with low SA, while participants 1 (85.33), 4 (85.42), 6 (85.21), 10 (89.91), and 11 (89.67) belonged to the group with high SA.

### 3.2 EEG status

**Figure 7:**
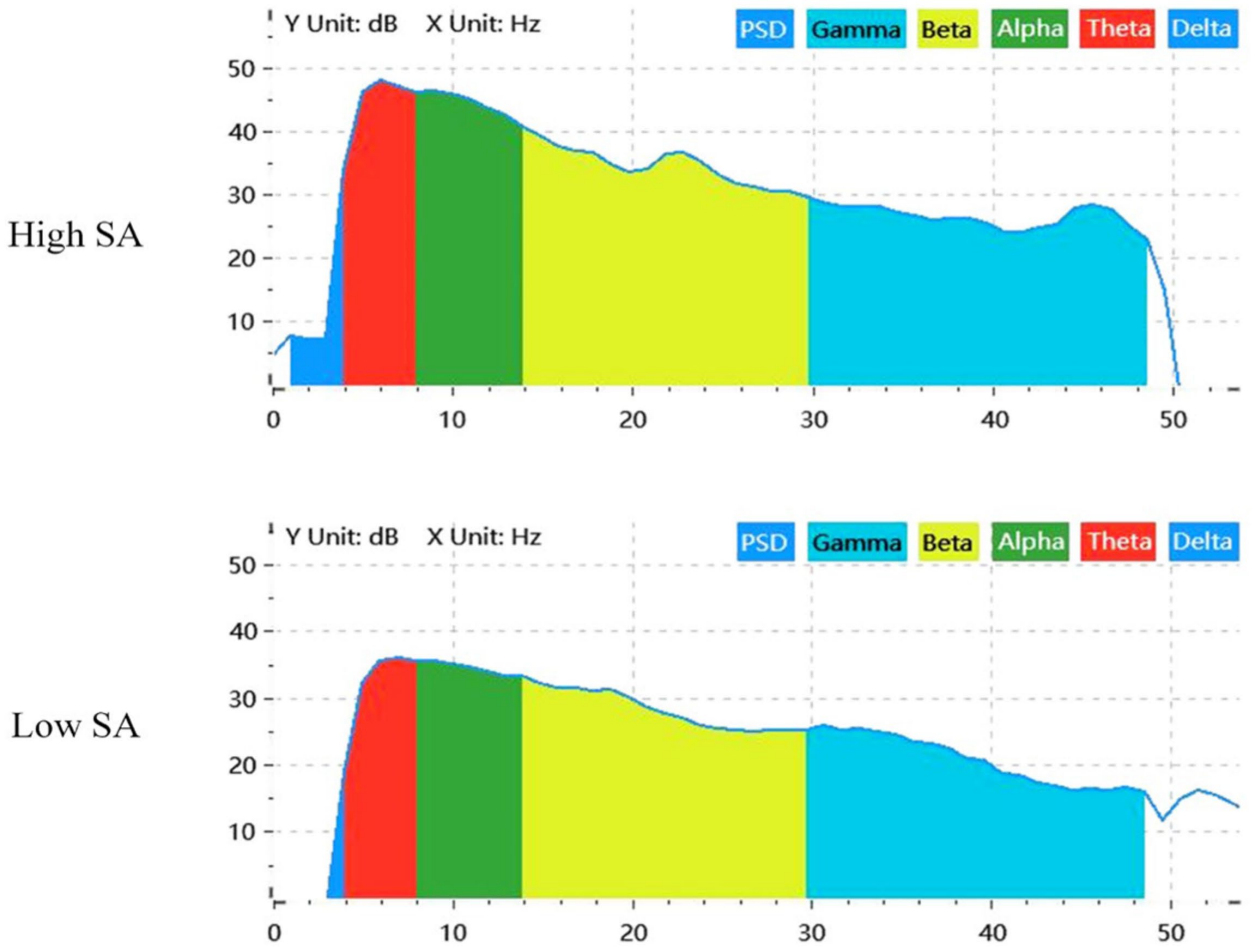
PSD characteristics of EEG for different SA groups at fixed time periods. SA, situation awareness; PSD, power spectral density.

Comparing the PSDs of the two SA groups, the total power of the EEG signal in the high SA group was higher than that of the low SA group in all frequency bands, and the maximum power in the high SA group was also higher than that of the low SA group (48.10 dB > 36.12 dB), indicating that the participants in the high SA group had a higher level of brain activity, as shown in Figure 7. Specifically, the total percentages of *θ*, *α*, and *β* bands accounted for more than 90% in both SA groups, indicating that the three bands were relatively active; and there were significant differences in the percentages of these three bands in different SA groups, as listed in Table 2. These results provide an opportunity for subsequent SA identification studies to combine *θ*, *α*, and *β* bands.

**Table 2:** PSD analysis of different frequency bands for two SA groups.

Frequency band	High SA group			Low SA group		
	Total power (dB)	Average power (dB)	Power percentage (%)	Total power (dB)	Average power (dB)	Power percentage (%)
*δ*	19.23	7.54	0.01	4.12	0.62	0.01
*θ*	52.08	46.06	36.47	39.73	33.71	20.90
*α*	53.16	45.38	46.78	42.58	34.80	40.25
*β*	48.09	36.05	14.57	41.53	29.49	31.59
*γ*	39.84	27.05	2.18	35.13	22.35	7.25

The total power is the total energy value of the band, the average power is the average energy value of the band, and the power percentage is the percentage of the energy value of the band to the total energy value of all bands. It is worth noting that the total power in the table represents the average of the total power of all participants in each group, and the average power similarly represents the average of the average power of all participants in each group. PSD, power spectral density; SA, situation awareness.

**Figure 8:**
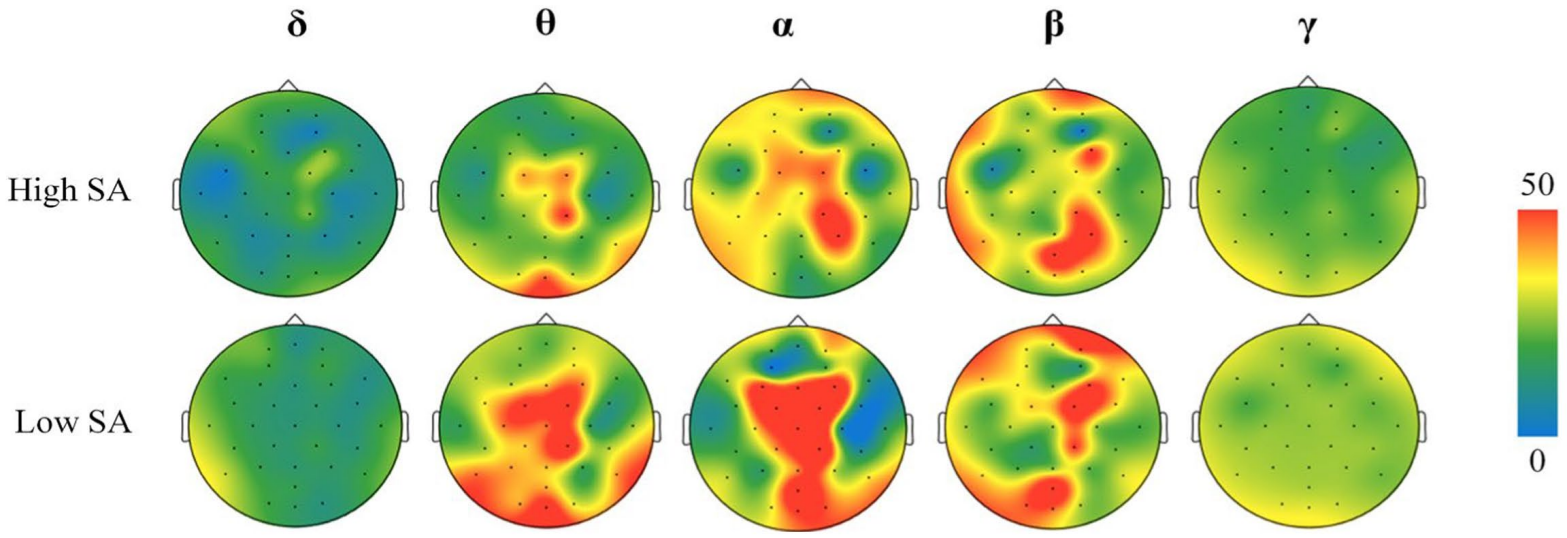
Frequency band activity maps of different brain regions in high and low SA groups. All participants’ brain topography maps are provided in Figure A1. SA, situation awareness.

Two participants with different SA levels were selected and their EEG data were visualized as brain topography during a fixed time period of the full-scene task, and the results are shown in Figure 8. It was found that the activation of *θ*, *α*, and *β* frequency bands in the brains of forklift drivers with different SA levels were greater throughout the task and were mainly located in four brain regions, F, C, P, and O. At the same time, there were significant differences between different SA groups in the activation of each frequency band, which showed the phenomenon of incomplete overlap in the distribution of each brain region. For example, in the θ frequency band, the high SA group was warmer and more active in areas C and O, whereas the low SA group was relatively active in all four brain areas. Relatively speaking, the *δ* and *γ* brainwave frequency bands were less active in each brain region of the different forklift drivers, and there were no significant differences.

Since the effects of extraneous factors such as fatigue and anxiety were excluded during the forklift task scenario experiment and no cross-modal sensory task processing was performed, the participants’ *δ* and *γ* band activation in different brain regions was not significant. In contrast, the degree of activation in the *θ*, *α*, and *β* frequency bands was greater and significantly different in the high and low SA groups, further validating the results of the PSD analysis.

**Figure 9:**
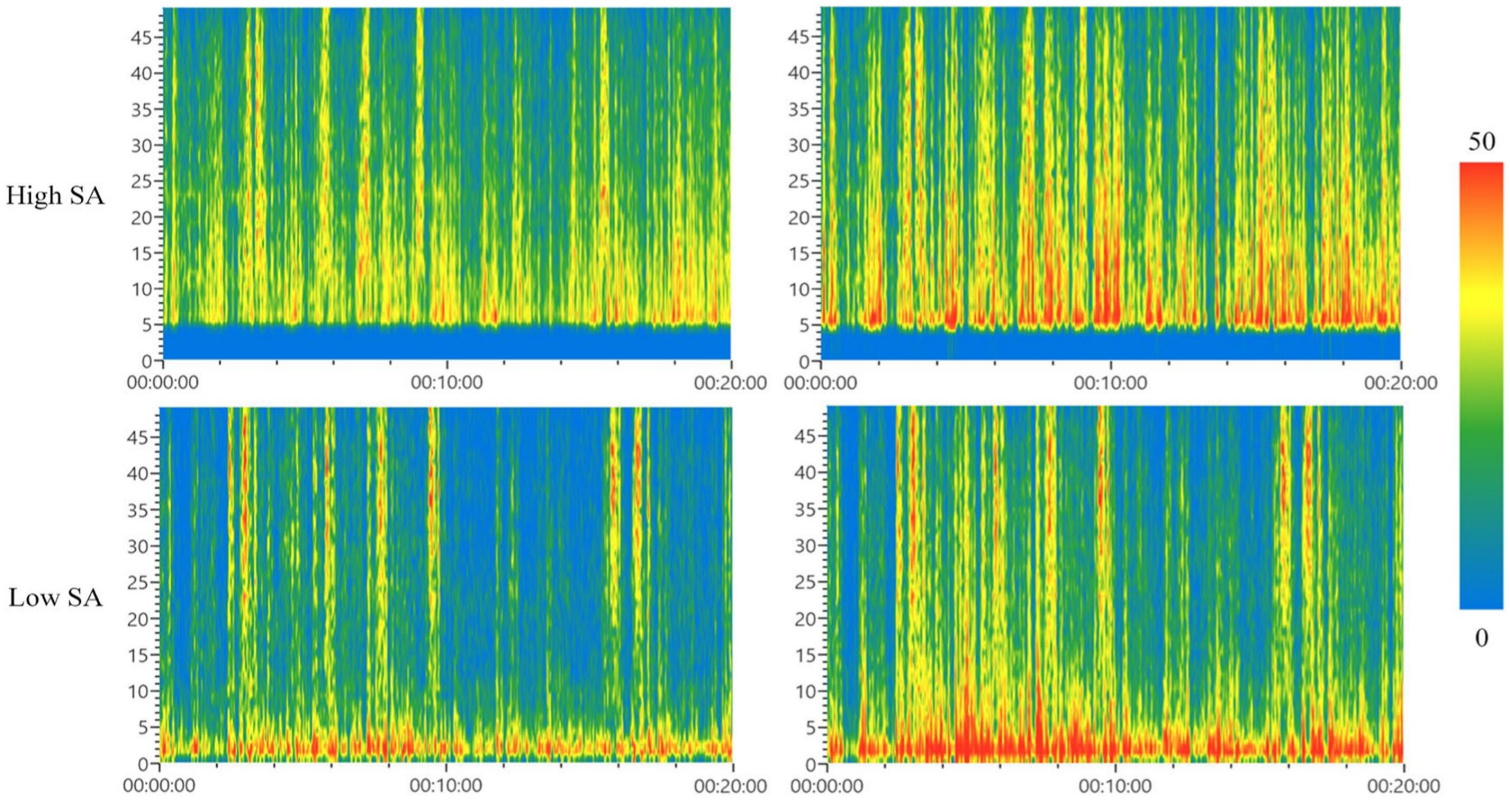
Time-frequency analysis diagrams of F-zone (left) and C-zone (right). SA, situational awareness.

**Figure 10:**
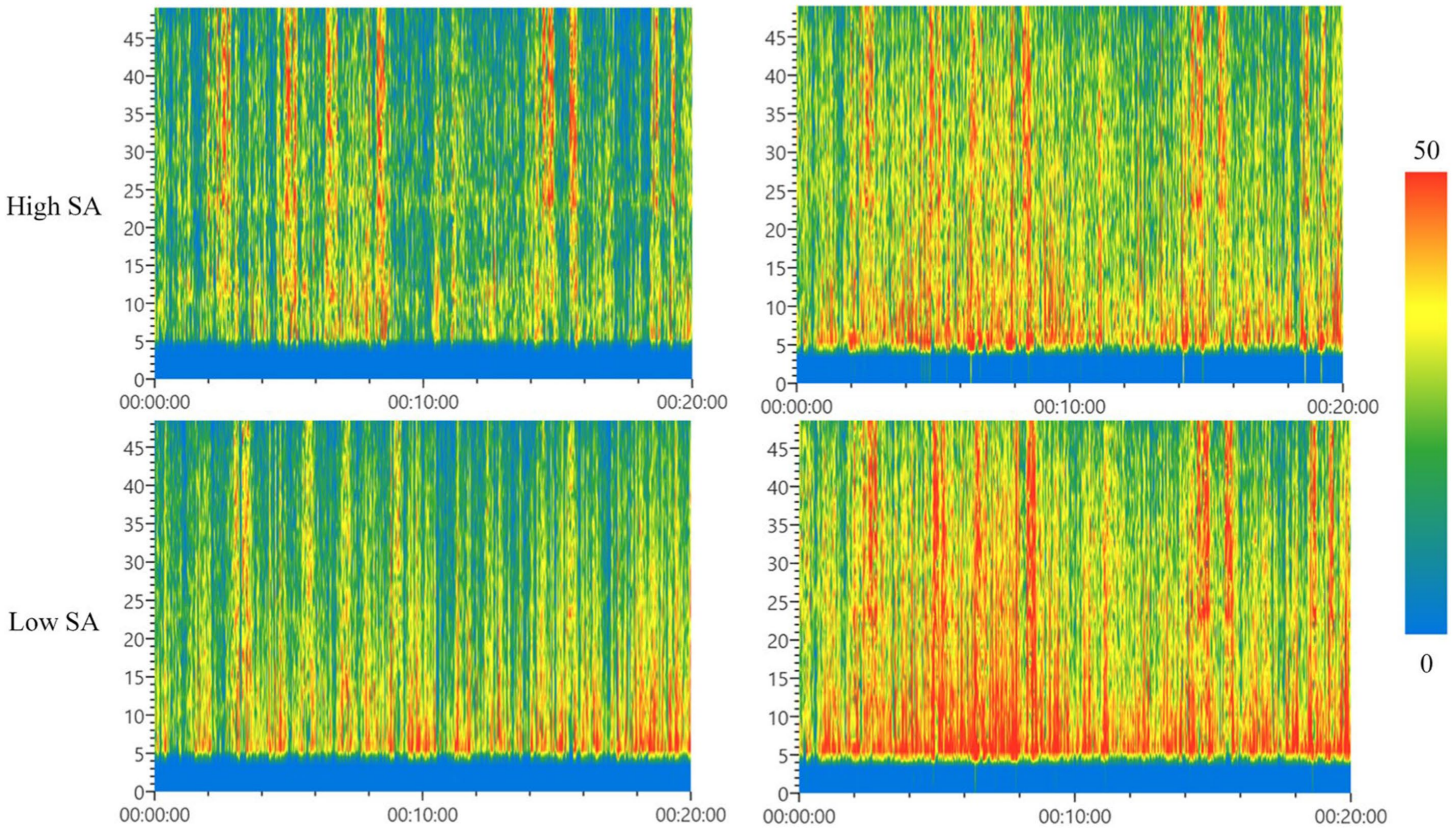
Time-frequency analysis of P-zone (left) and O-zone (right). SA, situation awareness.

The results of the above brain topography analysis showed that the signals of each frequency band of the forklift drivers’ EEG were mainly active in areas F, C, P, and O. Therefore, in order to reduce the redundancy of the EEG data analysis, time-frequency maps were drawn according to the same fixed time period (time length of 20 min) of the full-scene process in these four brain regions, and the results of the time-frequency analysis were obtained as shown in Figures 9, 10.

Figure 9 showed the time-frequency characteristics of forklift drivers in both high and low SA groups in area F (left) and area C (right). Comparing the left and right columns of the time-frequency plots, it was found that the warm peak columns in area C are significantly more than those in area F for forklift drivers with different SA levels, indicating that area C is more active than area F in terms of brain area energy; comparing the upper and lower plots, it was found that the time nodes of the appearance of warm peak columns in both areas F and C do not overlap, and there is a sequential. This indicated that the changes of brain wave energy in each frequency band in the two brain areas do not overlap with time, i.e., temporal difference between the high and low SA groups. The results of the analysis showed that there were significant differences in the changes of brain wave energy in different frequency bands in area F and area C in forklift drivers with different SA levels, in other words, there was variability in the instantaneous frequency characteristics.

Figure 10 showed the time-frequency characteristics of forklift drivers in both high and low SA groups in area P (left) and area O (right). Comparing the left and right plots, we found that the warm peak columns in area O were significantly richer than those in area P for both high and low SA forklift drivers, indicating that area O was more active than area P in terms of brain area energy. However, when comparing the upper and lower plots, it was found that the time nodes of the warm peak columns in area P of forklift drivers with different SA levels did not overlap and there was a sequential order, indicating that the changes in brain wave energy in each frequency band over time in the high and low SA groups did not overlap, i.e., there was a temporal difference. In contrast, the upper and lower plots of the warm peak column in the O area were almost identical, i.e., the changes in brain wave energy over time in each frequency band of the high and low SA groups in the O area were consistent. The results of the analysis showed that there were significant differences in the changes of brain wave energy in different frequency bands in the P area of forklift drivers with different SA levels, and there was variability in the instantaneous frequency characteristics, while the variability in the temporal frequency characteristics on the O area was not significant.

### 3.3 Correlation between EEG and SA

#### 3.3.1 Hypothesis testing

Due to the small sample size of the pre-processed EEG feature indicators that qualified for the parametric test, the correlation between the combined EEG indicators and SA was analyzed using the independent samples t-test method. The EEG feature data of the three task scenes were first categorized for the whole scene, and then five EEG combination indicators (4 × 5) for each of the four brain regions of F, C, O, and P areas of the whole scene were tested for their correlation with SA levels using the independent samples t-test method, where the initial hypothesis was that there was no significant difference in the distribution of EEG combination indicators for forklift drivers with different SA levels. The results of this test were presented in Table 3.

**Table 3:** Results of the correlation test between the combined EEG indices of sub-brain regions and SA levels in the whole scene.

Brain region	EEG combination indicator	Levene test		Means test			
		*F*	*P*	*t*	*P*	Mean difference	Standard error difference
F	*α*/*β*	14.426	< 0.01	1.472	0.151	0.055	0.038
	*θ*/*β*	0.288	0.595	2.298	0.028*	0.123	0.053
	(*α* + *θ*)/*β*	4.887	0.035	2.139	0.090	0.057	0.039
	(*α* + *θ*)/(*α* + *β*)	0.940	0.340	2.286	0.029*	0.096	0.042
	*θ*/(*α* + *β*)	0.131	0.719	2.361	0.025*	0.071	0.030
C	*α*/*β*	15.373	<0.01	2.913	0.107	0.096	0.083
	*θ*/*β*	0.761	0.390	3.232	0.003*	0.172	0.053
	(*α* + *θ*)/*β*	3.522	0.070	3.339	0.002*	0.268	0.080
	(*α* + *θ*)/(*α* + *β*)	1.099	0.303	3.416	0.002*	0.136	0.040
	*θ*/(*α* + *β*)	0.157	0.695	3.211	0.003*	0.976	0.030
P	*α*/*β*	6.084	0.019	2.712	0.101	0.045	0.031
	*θ*/*β*	2.936	0.097	2.706	0.011*	0.034	0.049
	(*α* + *θ*)/*β*	5.168	0.030	2.848	0.008*	0.217	0.080
	(*α* + *θ*)/(*α* + *β*)	3.259	0.081	2.748	0.010*	0.108	0.039
	*θ*/(*α* + *β*)	2.281	0.141	2.533	0.017*	0.076	0.029
O	*α*/*β*	4.601	0.040	0.950	0.349	0.011	0.012
	*θ*/*β*	0.004	0.952	1.720	0.095	0.039	0.023
	(*α* + *θ*)/*β*	0.048	0.829	1.595	0.121	0.049	0.030
	(*α* + *θ*)/(*α* + *β*)	0.030	0.864	1.579	0.125	0.027	0.017
	*θ*/(*α* + *β*)	0.005	0.943	1.704	0.098	0.026	0.014

*p ≤ 0.05. EEG, Electroencephalography; F, frontal lobe; C, central area; P, parietal lobe; O, occipital lobe.

The results of the analysis showed that in area F, the combined EEG indicators *θ*/*β*, (*α* + *θ*)/(*α* + *β*), and *θ*/(*α* + *β*) of forklift drivers were significantly correlated with SA levels in the full scenario with independent samples *t*-test *p*-values (*p* = 0. 028; *p* = 0.029; *p* = 0.025) less than 0.05, while *α*/*β*, (*α* + *θ*)/*β* did not show significant correlations (*p* = 0.151; *p* = 0.090); in region C, *θ*/*β*, (*α* + *θ*)/*β*, (*α* + *θ*)/(*α* + *β*), and *θ*/(*α* + *β*) were significantly correlated with SA (*p* = 0. 003; *p* = 0.002; *p* = 0.002; *p* = 0.003), while *α*/*β* was not significantly correlated with SA (*p* = 0.107); in region P, *θ*/*β*, (*α* + *θ*)/ β, (*α* + *θ*)/(*α* + *β*), and *θ*/(*α* + *β*) were significantly correlated with SA (*p* = 0.011; *p* = 0.008; *p* = 0.010; *p* = 0. 017), while the correlation between *α*/*β* and SA (*p* = 0.101) was not significant; in area O, none of the five combined EEG indicators showed a significant correlation with SA levels of forklift drivers (*p* = 0.349; *p* = 0.095; *p* = 0.121; *p* = 0.125; *p* = 0.098).

In summary, the hypothesis testing analysis indicated that some combined EEG indicators in areas F, C, and P were significantly correlated with SA levels of forklift drivers, revealing the correlative effect between EEG characteristics of forklift drivers and SA.

#### 3.3.2 Sparse principal component

The PCA method was used to analyze the principal components of the combined EEG indicators, and the eigenvalues, variance contribution rates, and cumulative variance contribution rates of each principal component were calculated, as shown in Table 4, to further confirm the relationship effect between EEG state and SA of forklift drivers.

**Table 4:** Results of principal component analysis.

Parameter	Principal component 1	Principal component 2	Principal component 3
Eigenvalue	14.68	2.249	1.261
Variance contribution rate	73.40%	11.24%	6.30%
Cumulative variance contribution rate	73.402%	84.647%	90.951%

Through principal component analysis, 20 data dimensions with 5 indicators in each of the original four brain regions were transformed into 3 principal components. The analysis results showed that 90.951% (>90%) of the original data information could be explained by three principal components, which helped to improve the EEG feature extraction. The principal component coefficients were returned to the original values, and the LASSO regression method was used for multiple regression analysis. The original three principal components were compressed into two principal components according to the sparse loadings, and the analysis results are shown in Table 5.

**Table 5:** Results of sparse principal component analysis.

Combined EEG indicator	Principal component 1	Principal component 3	LASSO analysis coefficients	Weight (%)
F-*α*/*β*	−0.637437614	0.250674537	−0.386763077	2.64553375
F-*θ*/*β*	−0.821519441	−0.062723655	−0.884243096	6.048392648*
F-(*α* + *θ*)/β	−0.762720164	0.133223576	−0.629496588	4.305877596
F-(*α* + *θ*)/(*α* + *β*)	−0.818846747	0.001834025	−0.817012722	5.588523972*
F-*θ*/(*α* + *β*)	−0.807153709	−0.126327642	−0.933481351	6.385191769*
C-*α*/*β*	−0.659487343	0.105273035	−0.554214308	3.790932337
C-*θ*/*β*	−0.805149188	−0.19514026	−1.000289448	6.842171988*
C-(*α* + *θ*)/*β*	−0.796462931	−0.086786063	−0.883248994	6.0415928*
C-(*α* + *θ*)/(*α* + *β*)	−0.805483275	−0.146135112	−0.951618387	6.509252579*
C-*θ*/(*α* + *β*)	−0.783433546	−0.245539267	−1.028972813	7.038371715*
P-*α*/*β*	−0.684543853	0.167336441	−0.517207412	3.537798059
P-*θ*/*β*	−0.80281058	−0.095956188	−0.898766768	6.147737356*
P-(*α* + *θ*)/*β*	−0.795126584	0.008729959	−0.786396625	5.379103988*
P-(*α* + *θ*)/(*α* + *β*)	−0.801140146	−0.043209629	−0.844349775	5.77551467*
P-*θ*/(*α* + *β*)	−0.784769893	−0.14305395	−0.927823843	6.34649333*
O-*α*/*β*	−0.57730199	0.431289319	−0.146012671	0.998754723
O-*θ*/*β*	−0.679866638	0.030224732	−0.649641906	4.443675438
O-(*α* + *θ*)/*β*	−0.711604884	0.192792708	−0.518812176	3.548774952
O-(*α* + *θ*)/(*α* + *β*)	−0.695902804	0.116937434	−0.57896537	3.960234357
O-*θ*/(*α* + *β*)	−0.637771701	−0.044383405	−0.682155106	4.666071974

*Weight > 5%. EEG, Electroencephalography; F, frontal lobe; C, central area; P, parietal lobe; O, occipital lobe.

**Figure 11:**
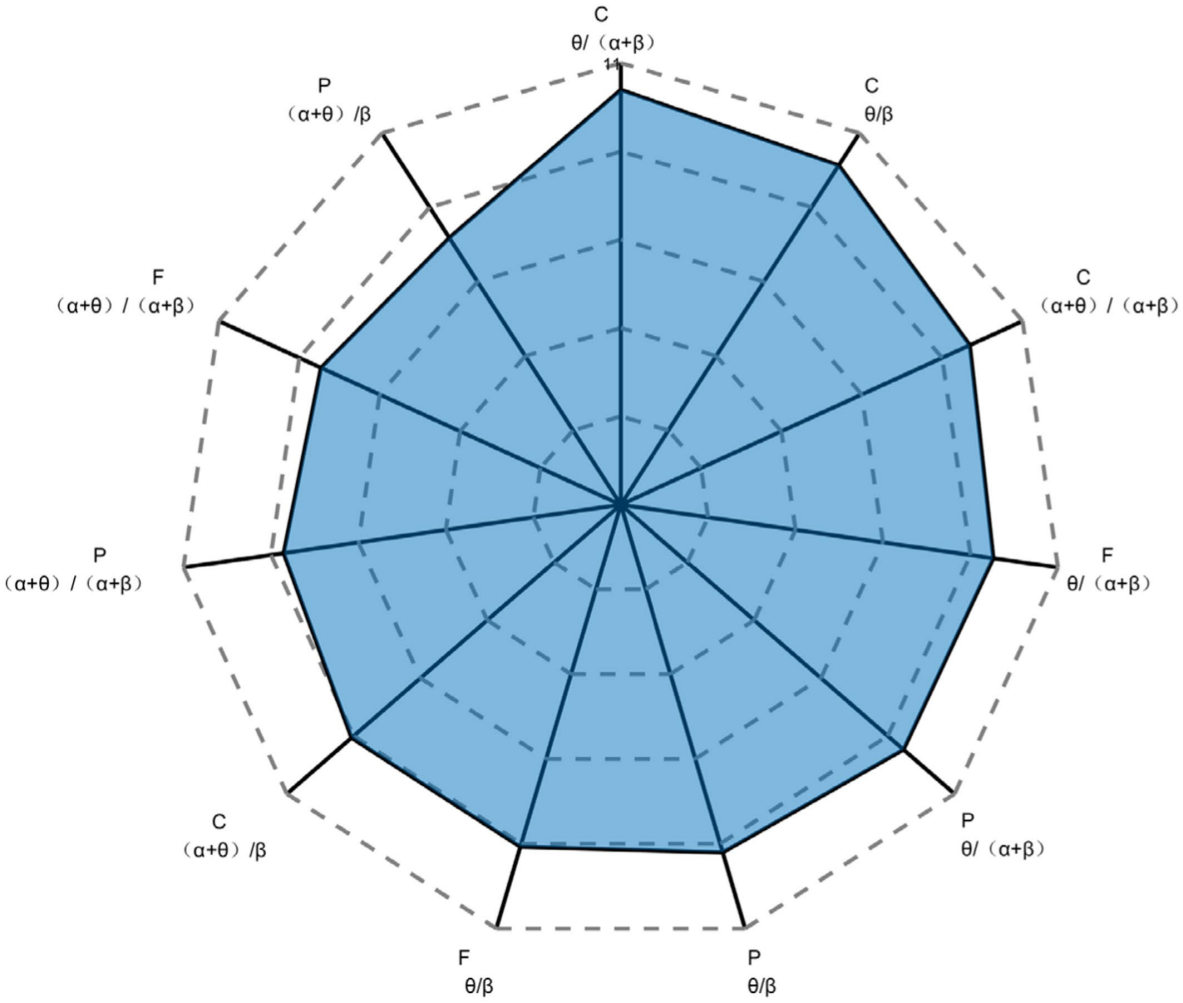
Sensitive EEG indicators of SA and their weights. SA, situation awareness; F, frontal lobe; C, central area; P, parietal lobe; O, occipital lobe.

The above 20 indicators were ranked according to their weight, and the EEG indicators with indicator component ratios greater than the mean (1/20) were extracted, which were 11 indicators in total. The comparative analysis revealed that the EEG indicators extracted by this method were completely consistent with the EEG indicators significantly correlated with SA obtained by the hypothesis testing method, as shown in Figure 11. Therefore, these 11 indicators were identified as sensitive EEG indicators of SA, i.e., as indicators that characterize the level of SA, for the next step of constructing a model to identify SA in forklift drivers.

### 3.4 SA recognition

#### 3.4.1 Model training

**Figure 12:**
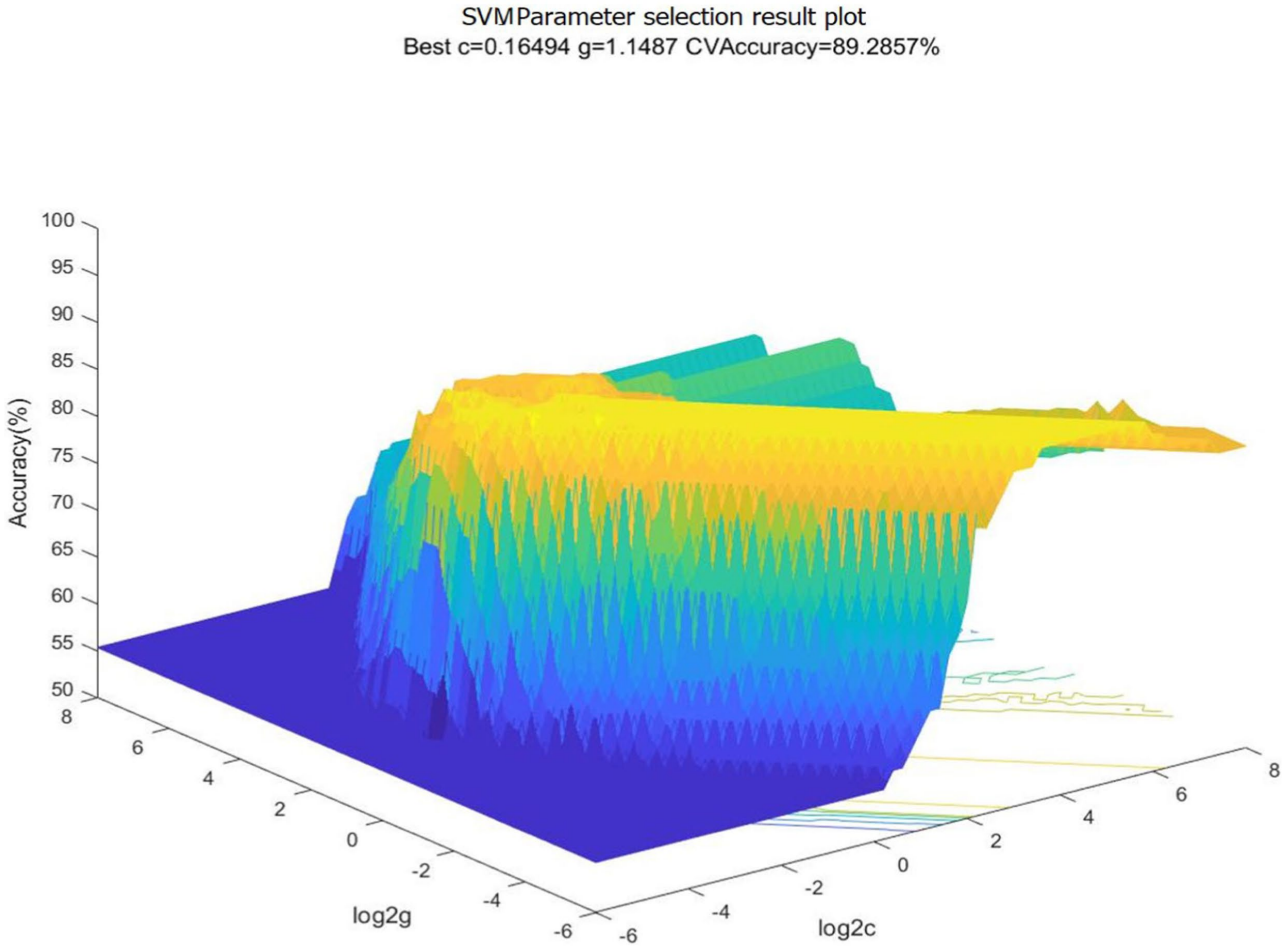
3D view of parameter selection results for SVM. c, penalty parameter; g, gamma, kernel function parameter.

**Figure 13:**
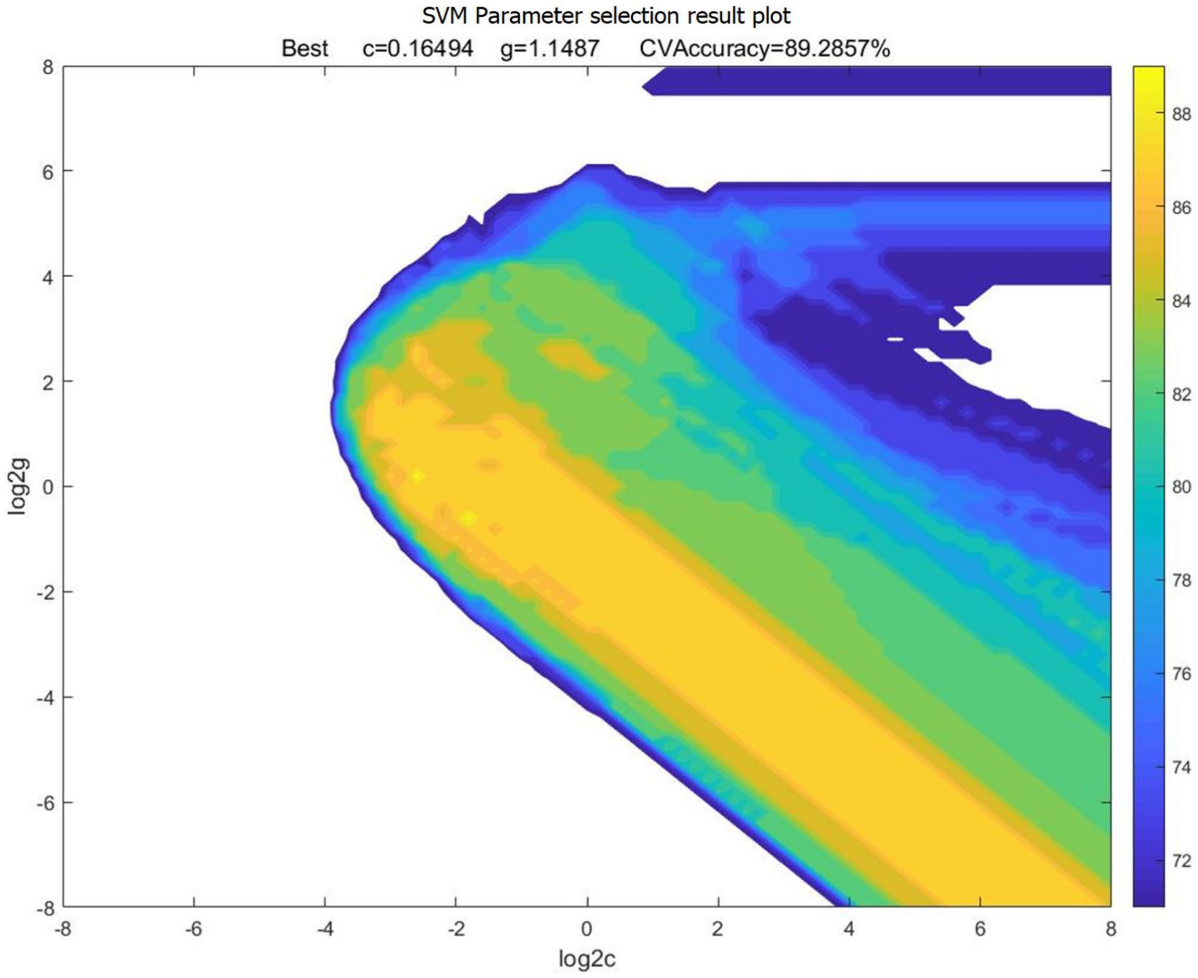
Contour map of parameter selection results for SVM. c, penalty parameter; g, gamma, kernel function parameter.

In this study, we extracted EEG data of forklift drivers with different SA levels using a time window of 10 s. After data extraction, a total of 110 sets of data were obtained, including 55 sets of EEG data from the high SA group and 55 sets of EEG data from the low SA group. For SVM recognition, 70% of the EEG data were selected as the training set, 15% of the EEG data were used as the validation set, and the remaining 15% of the EEG data were used as the test set. Meanwhile, to investigate the influence of different brain regions feature indicators on recognition accuracy, the 11 indicators obtained from the above correlation validation were combined according to the partition, forming a total of seven scenarios. The dataset of each scheme was trained, and the optimal parameters C, gamma corresponding to the SA recognition model of different schemes could be searched. Taking the one scheme of combined EEG indicators of area C & F & P as an example, the dataset of this scheme was input into the SVM model for training, and the optimal penalty parameter C = 0.16494 and the optimal kernel function parameter gamma = 1.1487 were found. The results of parameter selection were shown in Figures 12, 13.

#### 3.4.2 Model testing

The constructed SA recognition model was tested and evaluated using the recognition accuracy metric that is the ratio of the number of correct recognitions to the total sample size of the test set. In this study, 44 sets of EEG data of forklift drivers with different SA levels were randomly selected from the remaining dataset as the test set, and the SA level recognition results of the 44 sets were obtained after inputting the trained SVM model. The comparison with the SA levels of the forklift drivers in the test set found that the number of correctly identified groups was 39, while there were 5 incorrect groups. The recognition accuracy of the test set reached 88.64%, which verified the effectiveness of the model.

Based on the training results of the above three-zone EEG combination metrics, the SA recognition model was trained, tested, and evaluated for performance using datasets from other combination schemes in the interval of higher recognition accuracy. The recognition results are listed in Table 6.

**Table 6:** Accuracy of SA recognition with different EEG indicator combination schemes.

Combination of EEG indicators	Recognition accuracy (%)
F-zone	70.4527
C-zone	77.2709
P-zone	83.1818
F-zone and C-zone	79.6364
F-zone and P-zone	86.9091
C-zone and P-zone	84.9091
F-zone and C-zone and P-zone	88.6364

SA, situation awareness; EEG, Electroencephalography; F, frontal lobe; C, central area; P, parietal lobe; O, occipital lobe.

## 4 Discussion

The active state of forklift drivers’ EEG waves in each frequency band were found to be distributed nonlinearly in this study’s results of the EEG state analysis. The EEG signals in *θ*, *α*, and *β* frequency bands are active and mainly distributed in F, C, P, and O brain regions, which is consistent with the findings of many previous related studies (Fernandez Rojas et al., 2019; Kaur et al., 2020).

The correlation analysis verified the correlation between the combined EEG indicators of different frequency bands in different brain regions and SA. More specifically, the EEG indicators *θ*/*β*, (*α* + *θ*)/(*α* + *β*), and *θ*/(*α* + *β*) in regions F, P, and C are significantly correlated with SA, which has similarity to the band power ratio used to evaluate fatigue (Jap et al., 2009, 2011; Chen et al., 2013). However, the correlations of O-zone and indicator (*α* + *θ*)/*β* with SA levels were not further confirmed because they may be influenced by factors such as study objectives and experimental conditions. Finally, 11 combined EEG indicators: F-*θ*/*β*, F-(*α* + *θ*)/(*α* + *β*), F-*θ*/(*α* + *β*), C-*θ*/*β*, C-(*α* + *θ*)/*β*, C-(*α* + *θ*)/(*α* + *β*), C-*θ*/(*α* + *β*), P-*θ*/*β*, P-(*α* + *θ*)/*β*, P-(*α* + *θ*)/(*α* + *β*), and P-*θ*/(*α* + *β*), were extracted as characteristic indexes of SA level recognition for forklift drivers.

SVM was used to build a forklift driver SA recognition model based on these feature indicators. On the basis of brain regions, several feature indication combination schemes were created, and the model was trained, tested, and evaluated. The results showed that the model had the highest recognition accuracy of 88.64% with the combination of indicators in regions C & F & P, which exceeded the recognition accuracy of previous related studies (Kästle et al., 2021).

## 5 Conclusion

Aiming to objectively and accurately measure the SA of forklift drivers, this paper proposed an EEG identification method for SA of forklift drivers through field experiment design, variance analysis, correlation verification and model construction, testing and evaluation. The results verified the rationality and validity of the method. It can provide a new path for the study of SA theory and its measurement method and could help to improve the SA of forklift drivers and reduce the human errors in forklift operations.

However, due to the limitation of working time of forklift drivers and environmental conditions of the experimental field, the number of participants in this experiment is relatively small and the sample size is not sufficient. The experimental results need to be further validated. Furthermore, because of the limited space, other physiological indicators were not considered in this paper, and the assessment of forklift drivers’ SA under the data fusion of multidimensional physiological characteristics was not involved. This will be the direction of further research.

## Data availability statement

The raw data supporting the conclusions of this article will be made available by the authors, without undue reservation.

## Ethics statement

Ethical approval was not required for the studies involving humans because ethical review and approval was not required for the study on human participants in accordance with the local legislation and institutional requirements. The studies were conducted in accordance with the local legislation and institutional requirements. The participants provided their written informed consent to participate in this study.

## Author contributions

XL: Data curation, Investigation, Writing – original draft, Conceptualization, Methodology. YK: Formal analysis, Methodology, Writing – review & editing, Writing – original draft, Software. WC: Conceptualization, Funding acquisition, Project administration, Writing – review & editing. FL: Data curation, Visualization, Writing – review & editing. YJ: Resources, Supervision, Writing – review & editing. YL: Software, Validation, Writing – review & editing.

## Figures and Tables

**Figure A1:**
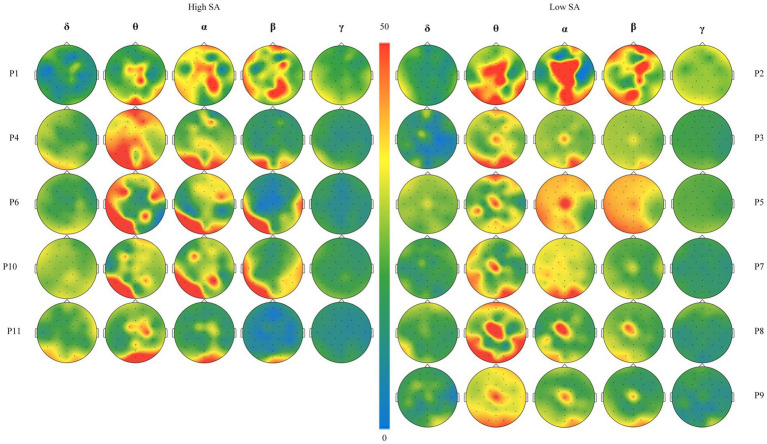
Frequency band activity map of different brain regions for all participants in high and low SA groups.
